# Study of enantioselective metolachlor adsorption by activated carbons†

**DOI:** 10.1039/d0ra07745c

**Published:** 2020-11-05

**Authors:** Alicia Gomis-Berenguer, Isabelle Laidin, Sophie Renoncial, Benoît Cagnon

**Affiliations:** Interfaces, Confinement, Matériaux et Nanostructures-ICMN, UMR 7374-CNRS, Université d'Orléans 1B, Rue de La Férollerie, CS 40 059 45071 Orléans Cedex 2 France aliciagb@kth.se benoit.cagnon@univ-orleans.fr; JACOBI Carbons 15 Route de Foëcy 18100 Vierzon France

## Abstract

Four activated carbons were employed to analyse the adsorption of different enantiomeric mixtures of the herbicide metolachlor in aqueous solution. The adsorption kinetics and isotherms were measured and fitted with different theoretical models to exhaustively analyse the adsorption mechanism. Different adsorption capacities were observed as a function of textural features of the adsorbents revealing an important effect of the presence of micro and mesoporous development on the adsorbent–adsorbate interactions. Additionally, enantioselective adsorption was detected for two of the activated carbons employed, rendering a greater adsorption of the *S*-metolachlor enantiomer compared to the racemic mixture. This fact was associated to the accessibility of certain conformers of the herbicide to the larger pores, facilitating the non-electrostatic adsorption.

## Abstract 2

Enantioselective adsorption of metolachlor. The adsorption of *S*-enantiomer is favored in certain activated carbons.

## Graphical abstract

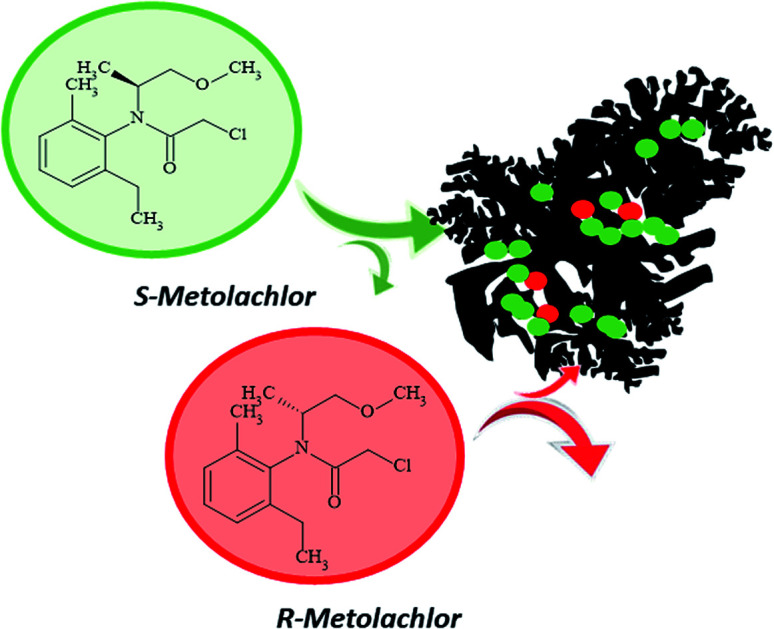

## 1. Introduction

The pollution caused by pesticides has greatly increased due to their widespread use in agriculture around the world. Pesticides are highly noxious, sometimes non-biodegradable and very mobile throughout the environment (water and soils). For the above reasons, the scientific community is focused on the removal of these toxic pollutants from aqueous solutions by different mechanisms such as photocatalysis,^1,2^ adsorption,^3–5^ and electrochemical processes.^6,7^ Specifically, adsorption is highly used in drinking water treatment plants due to its low cost, high removal efficiency and easy operation compared to other techniques.

Metolachlor is a very widely used herbicide for selective leaf weed control in more than 70 crops.^8^ It belongs to the acetamide class of chiral herbicides and contains *R*- (R-MET) and *S*- (S-MET) enantiomers (present in equal ratio for racemic metolachlor, Rac-MET). Moser *et al.*^9^ reported that the *S*-enantiomer presents highest herbicidal efficiency, while the *R*-enantiomer possesses a superior fungicidal activity. Consequently, in many countries the use of racemic herbicide was forbidden and replaced by *S*-metolachlor, particularly, in France the use of racemic metolachlor was banned on 2003.^10^ Since the rate of dissipation and soil binding of both enantiomers is the same,^11^ this led to a reduction in the doses applied by farmers, maintaining the biological performance, and is expected to result in lower concentrations of the residues in the environment. However, due to its extensive use, high water solubility, low vapour pressure, and long-life, metolachlor can be widely detected in surface water and groundwater compromising the water quality.^12^

Although R-MET and S-MET adsorption has been extensively studied,^3,5,13^ specific data for selective adsorption of each enantiomer are scarce. To the best of the authors' knowledge, this work analyses for the first time, the selective adsorption of both enantiomers employing three commercial activated carbons and one activated carbon elaborated from agricultural residues as adsorbents.

## 2. Experimental section

### 2.1 Materials

Rac-metolachlor and enriched *S*-metolachlor (60%) were purchased from Sigma-Aldrich, pure *S*-metolachlor (100%) was purchased from LGC Standards. For clarity, the physicochemical properties including the molecular structure of each enantiomer are shown in Table 1. The chemicals were used without further purification. All solutions were prepared with ultra-pure water obtained from Milli-Q water purification systems.

**Table 1:** Physicochemical properties of *R*- and *S*-metolachlor

	*R*-Metolachlor	*S*-Metolachlor
Molecular formulae	C_15_H_22_ClNO_2_	C_15_H_22_ClNO_2_
IUPAC name	2-Chloro-*N*-(2-ethyl-6-methylphenyl)-*N*-[(2*R*)-1-methoxypropan-2-yl]acetamide	2-Chloro-*N*-(2-ethyl-6-methylphenyl)-*N*-[(2*S*)-1-methoxypropan-2-yl]acetamide
CAS number	178961-20-1	87392-12-9
Molecular weight (g mol^−1^)	283.79	283.79
Water solubility at 25 °C (mg L^−1^)^14^	480	480
Molecular size^a^ (nm)	0.98 × 0.78 × 0.41	0.98 × 0.78 × 0.41
Molecular structure	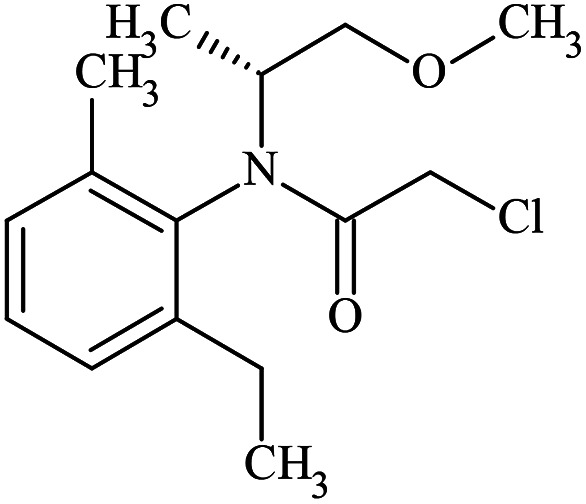	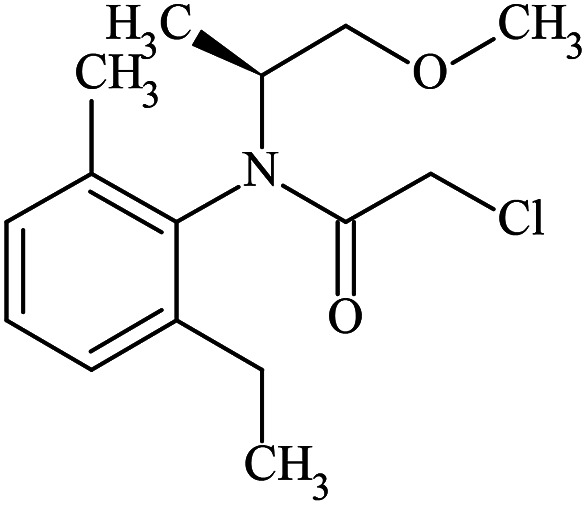

a Estimated from Chemdraw software after 3D optimization for the lowest energy configuration.

Three commercial granular activated carbons (L27, AQ630 and S21) were supplied by Jacobi Carbons (Vierzon, France). The materials were grinded to obtain a particle size *ca.* <80 μm and washed with distilled water until constant pH to remove the residual activating agent. Additionally, an activated carbon obtained by chemical activation from a lignocellulosic precursor (rape straw) was also employed. The material (named as R-KC) was synthesized by chemical activation, briefly 1 g of rape straw powder was physically impregnated with 1 g of K_2_CO_3_ in a mortar, then the activation was performed at 800 °C for 1 h under N_2_ flow of 160 mL min^−1^ (heating rate 10 °C min^−1^) in an horizontal furnace. After cooling under a N_2_ flow, the material was thoroughly washed with distilled water until constant pH to remove any water-soluble species and the excess of oxidising agent, dried at 60 °C overnight and stored in a desiccator.

### 2.2 Characterization of the adsorbents

The porosity of the materials was characterized by measuring the N_2_ adsorption/desorption isotherms at 77 K (ASAP 2020, Micromeritics). The materials were previously outgassed under vacuum (*ca.* 10^−4^ Pa) at 120 °C for 17 h. The isotherms were used to determine several textural parameters as specific surface area (*S*_BET_), specific total pore volume (*V*_total_), specific micro- and meso-pores volumes (*W*_0_, *W*_meso_) and mean micropore size (*L*). The surface pH was determined measuring the pH of an equilibrated aqueous suspension (*ca.* 1 g L^−1^) of the adsorbent. Elemental analysis was carried out by Thermo Scientific FLASH 2000 automatic analyser. TEM (transmission electron microscopy) images were obtained by a microscope (Philips CM20, Philips Co. Ltd.) operating at 200 kV.

### 2.3 Adsorption experiments

To study the adsorption kinetics experiments, a solution volume of 100 mL of each compound with an initial concentration of 25 mg L^−1^ was mixed with 5 mg of adsorbent (0.5 mg compound per mg adsorbent) in glass flasks and continuously stirred (400 rpm) on a multi-stirring plate (IKA brand) at 25 °C in a thermostatic water bath. Aliquots were measured at specific time intervals until equilibrium was reached. Solutions were filtered through a Nylon syringe filter (0.45 μm) and the concentration was determined by reverse-phase HPLC in an apparatus equipped with a photodiode array detector (Shimadzu Nexera XR). A C-18 column (Restek) with a particle size of 2.7 μm (3 mm × 100 mm) was employed. As a mobile phase water : acetonitrile (40 : 60 v/v) was used at a flow rate of 0.8 mL min^−1^. The column was thermostated at 30 °C and the sample injection volume was 50 μL. Both metolachlor enantiomers (*R*- and *S*-) were detected at wavelength of 230 nm after 5.5 min (no chiral separation was produced). An example of racemic metolachlor's chromatogram is shown in Fig. S1 ESI.† LabSolutions software from Shimadzu was used for identification and quantification of the molecule. The concentration of the compound was calculated based on the relative peak areas using standards of known concentrations. Equilibrium adsorption isotherms were also carried out from 25 mL of solutions of initial concentration ranging from 20 to 150 mg L^−1^ with 2.5 mg of adsorbent (0.2–1.5 mg compound per mg adsorbent) during 24 h (equilibrium time based on the kinetic study). All adsorption assays were made by duplicate and a blank solution (without adsorbent) was used to check the adsorption of the sorbate on the walls.

The amount of compound adsorbed was determined following the equation:^15^
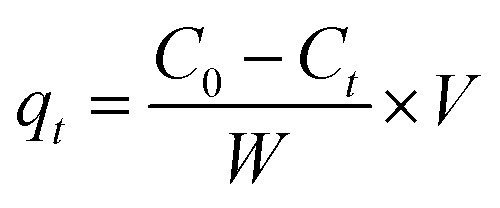
where *q*_*t*_ is the amount adsorbed (mg g^−1^) at time *t*, *C*_0_ and *C*_*t*_ are the compound concentrations (mg mL^−1^) in the initial solution and at time *t* respectively, *V* is the volume (mL) of the solution and *W* is the weight (g) of adsorbent. The removal efficiency percentage was calculated by:
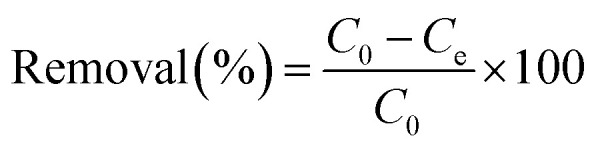
where *C*_0_ and *C*_e_ are the concentration of the compound before and after adsorption respectively.^15^

### 2.4 Data modelling analysis

Lagergren pseudo-first order^16^ and Ho's pseudo-second order^17,18^ models were applied to describe the kinetics adsorption of the molecules. The values of rate constants and the amounts of adsorption at equilibrium were determined by the non-linear method using Origin software. The pseudo-first order kinetic equation is expressed as:*q*_*t*_ = *q*_e_(1 − e^−*k*_1_*t*^)where *k*_1_ is the rate constant of pseudo-first order adsorption in (h^−1^), *q*_e_ is the amount of solute adsorbed at equilibrium in (mg g^−1^) and *t* the time in (h).

The pseudo-second order kinetic equation is expressed as follows:
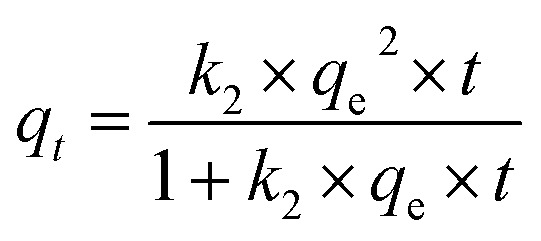
where *k*_2_ is the rate constant of pseudo-second order adsorption in (g mg^−1^ h^−1^).

The adsorption mechanism was analysed applying to the data the Morris–Weber equation:^19^
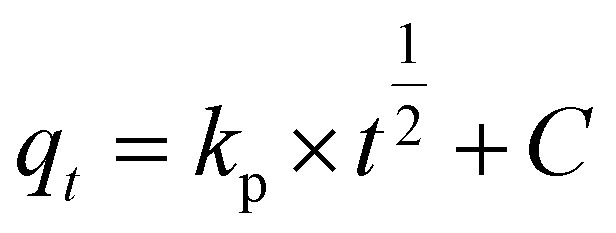
where *q*_*t*_ is the amount adsorbed at time *t* (mg g^−1^), *k*_p_ is the rate constant of intraparticle diffusion (mg g^−1^ h^−1/2^) and *C* is the intercept which is related to boundary layer thickness.

The equilibrium experimental adsorption data were fitted to Langmuir, Freundlich and Dubinin–Radushkevich–Kaganer (DRK) models.

Langmuir equation describes the monolayer adsorption and allows the calculation of the maximum amount adsorbed to complete the monolayer (*q*_max_ in mg g^−1^).^20^ Its expression is:
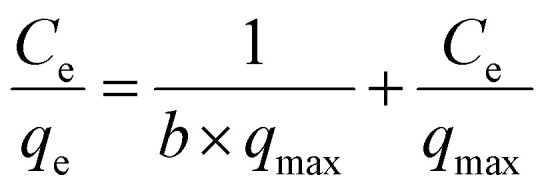
where *C*_e_ is the liquid phase solute equilibrium concentration (mg L^−1^), *q*_e_ is the adsorbed amount of solute per unit gram of adsorbent (mg g^−1^) and *b* (L mg^−1^) is the Langmuir adsorption constant which is related to the energy of adsorption.

Freundlich model^21^ is an empirical model and assumes that the adsorption occurs on a heterogeneous surface. It is described as:*q*_e_ = *k*_f_ × *C*_e_^1/*n*_f_^where *k*_f_ and *n*_f_ are Freundlich constants and are dependent on the solute–adsorbent interaction.

DRK model^22^ is described by the equation:
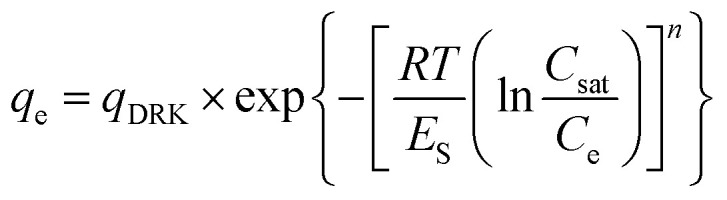
where *T* is the temperature and *R* is the gas constant. Considering *n* = 4 and the *C*_sat_ equal to the solubility in water of the solute (mg L^−1^), it is possible to determine the *q*_DRK_ which is the limiting amount filling the micropores at equilibrium (mol g^−1^) and *E*_s_ that is the characteristic energy of adsorption (J mol^−1^).^22^

## 3. Results and discussion

### 3.1 Textural and chemical characterization of adsorbents

**Fig. 1:**
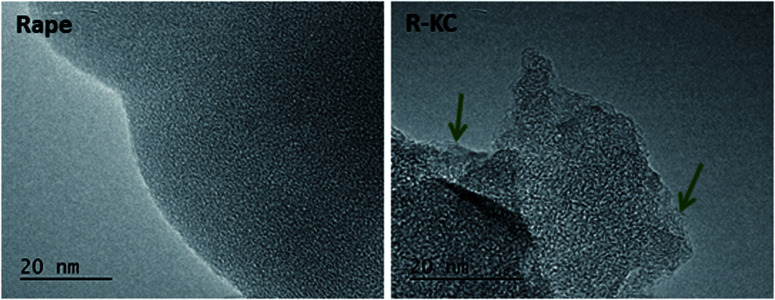
TEM images of rape straw (left) and R-KC material (right).

Fig. 1 depicts the TEM images of the R-KC adsorbent and its precursor (rape straw). The raw material presents a homogeneous amorphous structure and the R-KC image reveals that the chemical activation process by K_2_CO_3_ provokes a slightly expansion of the structure with formation of small ordered regions (indicated by arrows in the figure).

**Fig. 2:**
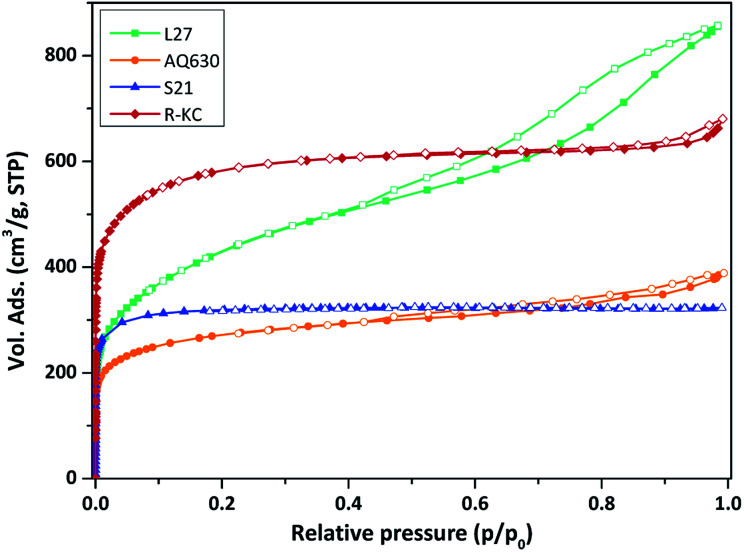
Nitrogen adsorption/desorption isotherms at 77 K of the studied adsorbents. Close symbols represent adsorption and empty symbols represent desorption.

Fig. 2 shows the N_2_ adsorption/desorption isotherms obtained at 77 K for all the adsorbents. The four activated carbons are clearly different in terms of porous properties. AQ630, S21 and R-KC samples present a Type I(a) isotherm according to the IUPAC classification,^23^ characteristic of microporous materials, while, L27 shows a Type IV isotherm with a prominent hysteresis loop above 0.4 of relative pressure, typical for micro-mesoporous materials. The highest surface area was obtained for R-KC (2200 m^2^ g^−1^) that results twice the value of AQ630 (Table 2). The micropore volume was found between 0.38 and 0.83 cm^3^ g^−1^ following the trend: R-KC > L27 > S21 > AQ630. According to the isotherm, sample L27 shows the highest mesoporous development with a volume of 0.79 cm^3^ g^−1^; for samples AQ630 and R-KC similar mesoporous volumes were obtained (*ca.* 0.20 cm^3^ g^−1^) and S21 does not show the presence of mesopores, it is only microporous.

**Table 2:** Textural parameters obtained from the N_2_ isotherms at 77 K, chemical composition obtained from elemental analysis (wt%) and surface pH of all the adsorbents

Sample	*S*_BET_ (m^2^ g^−1^)	*V*_total_ a (cm^3^ g^−1^)	*W*_0_ b (cm^3^ g^−1^)	*L* c (nm)	*W*_meso_ d (cm^3^ g^−1^)	*W*_meso_/*W*_0_	*C* (wt%)	N (wt%)	O (wt%)	O/C	Surface pH
L27	1573	1.350	0.532	2.20	0.793	1.49	89.1	<0.02	5.2	0.06	5.68
AQ630	1016	0.600	0.382	1.20	0.195	0.51	84.7	0.10	2.3	0.03	7.37
S21	1266	0.500	0.455	0.93	0.000	0.00	93.1	<0.02	2.2	0.02	7.34
R-KC	2220	1.047	0.832	1.23	0.130	0.16	86.4	<0.02	6.6	0.08	6.55

a Evaluated at *p*/*p*_0_ ∼ 0.99.

b Evaluated by DR method.

c Evaluated by Stoeckli–Ballerini equation.

d Evaluated by the 2D-NLDFT-HS method.

Chemical composition of the adsorbents was evaluated by elemental analysis (Table 2) showing important differences on the O amounts that were higher for samples L27 and R-KC, this is in concordance with the surface pH (and thermogravimetric profiles, Fig. S2 ESI†) that reveals the slightly acidic character for these two adsorbents, while the materials AQ630 and S21 presented neutral-basic character.

### 3.2 Adsorption of Rac-metolachlor: kinetics and isotherms

**Fig. 3:**
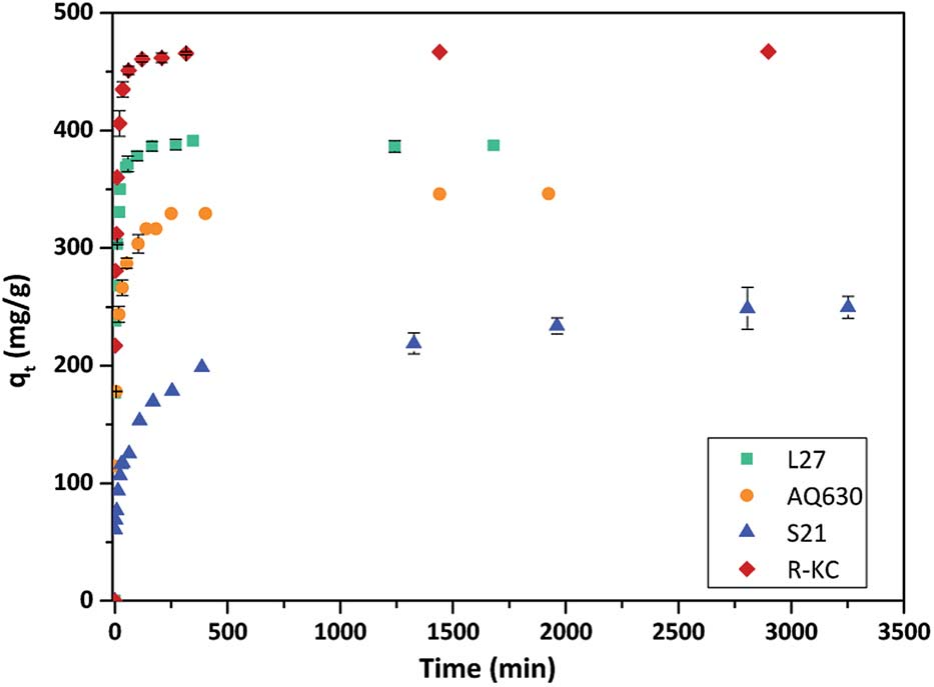
Evolution of Rac-metolachlor adsorption as a function of the contact time at 25 °C for the four adsorbents. The error bars represent the standard deviation.

Fig. 3 and S3 ESI† show the rate of the Rac-metolachlor adsorption on the four studied materials; in all cases the same concentration (25 mg L^−1^) and adsorbent amount (5 mg) were employed. An increase in the adsorption time implies an increase in the removal of Rac-MET before equilibrium was reached. Similar kinetic profiles were obtained regardless the nature of the carbonaceous adsorbent, although the uptake at equilibrium conditions was very different for each adsorbent. For the AQ630, L27 and R-KC carbon materials, the adsorption was relatively fast, reaching the equilibrium after *ca.* 3 h, however the equilibrium for S21 was reached after *ca.* 6 h. This fact can be associated to the absence of mesopores, since its presence facilitates the adsorption of the molecule during the first steps – they are able to provide a short path for the molecule diffusion inside the porosity of the activated carbon (from the mesopores to the micropores).^24^ The experimental data were fitted by a non-linear pseudo-first and pseudo-second order kinetic models.^16–18^ As shown in Table 3, the correlation coefficients, *R*^2^, for the pseudo-second order kinetics model (0.882–0.995) were best fitted than those for the pseudo-first order model (0.777–0.954), additionally, the calculated *q*_e_ values are closer to the experimental ones indicating that the adsorption mechanism is governed by the pseudo-second order regime for all the studied adsorbents. The highest value of the rate constant was obtained for the adsorption on L27 (0.055 g mg^−1^ h^−1^), while similar values were obtained for AQ630 and R-KC (0.034 and 0.047 g mg^−1^ h^−1^, respectively). This confirms that the presence of the mesopores (transport pores) increases the adsorption rate, since L27 presents the higher mesoporous development while AQ630 and R-KC show similar mesoporous volume. S21 has no development of mesopores showing the lower rate constant (0.010 g mg^−1^ h^−1^). The highest adsorption capacity was obtained for R-KC (reaching 99% of adsorption at equilibrium time) followed by L27, AQ630 and S21 (Fig. 3, S3 ESI† and Table 3). These differences could be associated to the different textural parameters of the adsorbents, particularly in their micro and mesoporous networks. Furthermore, the pore size (Table 2) has an important effect on the adsorption process since the activated carbon with lower pore size (S21) presents the lower adsorption.

**Table 3:** Pseudo-first and pseudo-second order Rac-MET, S-MET (60%) and S-MET (100%) adsorption parameters for the studied adsorbents obtained by a non-linear fitting. The coefficient of determination (*R*^2^) is also included

Molecule	Adsorbent	*q*_e, exp_ (mg g^−1^)	Removal efficiency at equilibrium (%)	Pseudo-first order			Pseudo-second order			Intraparticle model		
				*k*_1_ (h^−1^)	*q*_e_ (mg g^−1^)	*R* ^2^	*k*_2_ (g mg^−1^ h^−1^)	*q*_e_ (mg g^−1^)	*R* ^2^	*k*_p_ (mg g^−1^ h^−1/2^)	*C*	*R* ^2^
Rac-MET	L27	390 ± 5	85 ± 1	12.95 ± 0.50	371 ± 16	0.944	0.055 ± 0.003	388 ± 18	0.994	355 ± 18	133 ± 6	0.952
	AQ630	340 ± 5	77 ± 2	7.79 ± 0.50	314 ± 18	0.928	0.034 ± 0.002	330 ± 15	0.983	220 ± 9	98 ± 4	0.915
	S21	240 ± 16	51 ± 7	1.62 ± 0.10	207 ± 9	0.777	0.010 ± 0.000	222 ± 10	0.882	58 ± 3	64 ± 3	0.954
	R-KC	465 ± 2	99 ± 0.5	13.02 ± 0.70	448 ± 25	0.954	0.047 ± 0.002	467 ± 24	0.995	357 ± 17	184 ± 8	0.917
S-MET (60%)	L27	369 ± 2	88 ± 0.5	16.98 ± 0.90	351 ± 19	0.927	0.077 ± 0.003	364 ± 19	0.984	279 ± 14	145 ± 6	0.901
	AQ630	405 ± 7	84 ± 2	9.30 ± 0.30	382 ± 21	0.900	0.038 ± 0.003	395 ± 16	0.971	209 ± 11	172 ± 8	0.938
	S21	275 ± 11	52 ± 4	1.29 ± 0.10	231 ± 15	0.817	0.008 ± 0.000	245 ± 12	0.902	74 ± 3	55 ± 2	0.918
	R-KC	498 ± 2	98 ± 0.5	33.90 ± 2.00	467 ± 21	0.936	0.088 ± 0.005	497 ± 20	0.986	457 ± 22	200 ± 9	0.923
S-MET (100%)	L27	485 ± 3	96 ± 0.5	12.49 ± 0.50	425 ± 16	0.887	0.042 ± 0.002	451 ± 25	0.944	54 ± 3	343 ± 15	0.956
	AQ630	505 ± 10	96 ± 2	18.23 ± 1.00	476 ± 24	0.971	0.093 ± 0.004	487 ± 21	0.989	198 ± 5	340 ± 12	0.818
	S21	245 ± 9	52 ± 4	4.56 ± 0.30	182 ± 11	0.645	0.034 ± 0.001	195 ± 12	0.768	67 ± 2	78 ± 4	0.989
	R-KC	475 ± 1	99 ± 0.2	17.32 ± 1.00	466 ± 27	0.989	0.061 ± 0.002	485 ± 20	0.996	431 ± 22	195 ± 10	0.880

The adsorption mechanism of the Rac-MET on the four activated carbons was also analysed applying the intraparticle diffusion model. For all adsorbents, the *q*_*t*_*vs. t*^1/2^ plots exhibited a multi-linearity character (Fig. S4 ESI†), revealing the existence of two steps in the adsorption process. For the first linear stage, the adsorption of Rac-MET involves intraparticle diffusion (diffusion into the pores of the adsorbent), however, any curve passed through the origin, indicating that the rate-determining step is governed by several sorption mechanisms.^25^ The second stage corresponds to the final equilibrium step marked by an almost constant *q*_*t*_, due to the low Rac-MET concentration in solution. The rate constant, *k*_p_, calculated from the first stage (Table 3) may classified as follows: *k*_p_(R-KC) ∼ *k*_p_(L27) > *k*_p_(AQ630) > *k*_p_(S21). The smallest value for the S21 indicates that this activated carbon presents the lowest intraparticle diffusion rate that can be justified by the absence of mesopores.

**Fig. 4:**
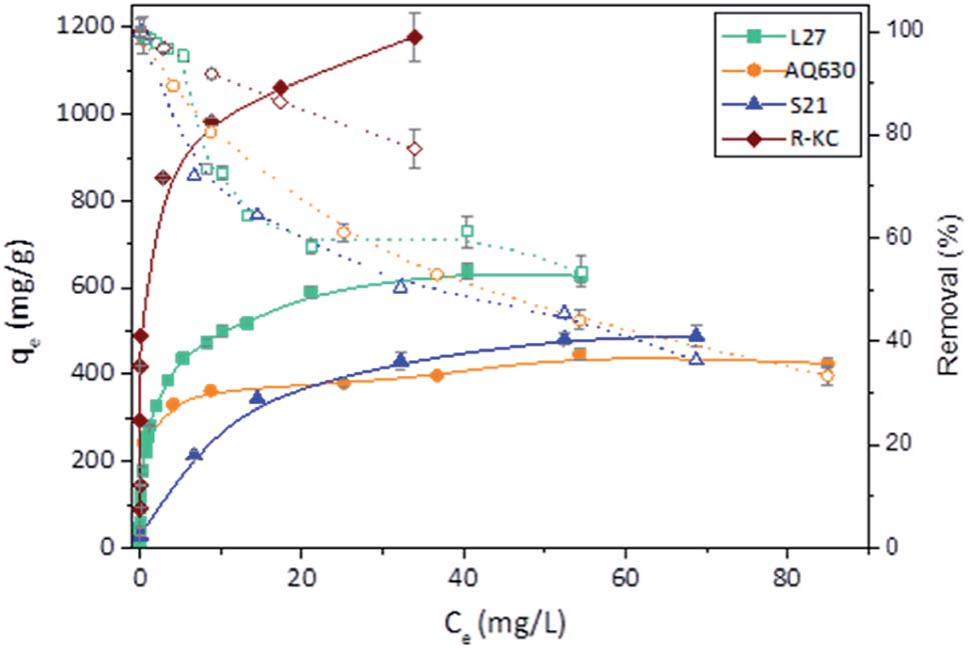
Rac-metolachlor experimental isotherms (filled symbols) and percentage removal (open symbols) of the four adsorbents at 25 °C. The error bars represent the standard deviation.

Further information about the adsorption mechanism can be obtained analysing the adsorption isotherms (Fig. 4). According to the Giles classification,^26^ all the adsorbents presented a L type isotherm, concave to the concentration axis with a defined plateau. This type of adsorption isotherms is associated to a monolayer adsorption with no strong competition of the solvent and without interactions between adsorbed molecules. The adsorption capacity values were found similar for AQ630 and S21 (423 and 485 mg g^−1^, respectively) and higher values were obtained for L27 (633 mg g^−1^) and R-KC (1178 mg g^−1^). These differences are mainly associated to the textural development of the activated carbons, being the material with the highest surface area (R-KC, Table 2) the adsorbent with greater adsorption capacity. Additionally, S21 showed the less steep slope at low concentration range suggesting a weaker adsorption under low amount of solute. These adsorption isotherms point out the importance of the combining presence of micro and mesopores to favour the adsorption.

The experimental isotherms were fitted to theoretical Langmuir, Freundlich and Dubinin–Radushkevich–Kaganer (DRK) models, the fitted parameters are collected in Table 4. The regression coefficient values revealed similar goodness of fitting for Freundlich model (*R*^2^ between 0.95 and 0.98) for all the adsorbents. With the exception of S21, the values of amount adsorbed to complete the monolayer obtained by Langmuir model (*q*_max_) are close to the experimental amount adsorbed at equilibrium values. Furthermore, the fitting of the experimental data to the DRK model revealed *q*_DRK_ values (theoretical adsorption capacity on the micropores) slightly lower to the *q*_e,exp_ with the exception of S21 (purely microporous material) indicating that the adsorption of the Rac-MET takes place in both micro and mesoporous surface. Additionally, the calculation of the occupied microporous volume, by the equation:^22^*W*_0(occ)_=(q_DRK_ × *V*_molar_)/*W*_0_, showed high percentage of occupied microporous surface being 96, 95, 99 and over 100% for L27, AQ630, S21 and R-KC. This fact reveals that the four studied adsorbents present an accessible microporosity for the adsorption of Rac-MET and confirms that the differences on the experimental adsorption capacities between them are related to the presence of mesopores.

**Table 4:** Parameters obtained by non-linear fitting to Langmuir, Freundlich and Dubinin–Radushkevich–Kaganer (DRK) isotherms for Rac-MET, S-MET (60%) and S-MET (100%) on studied carbonaceous adsorbents

Molecule	Adsorbent	*q*_e,exp_ (mg g^−1^)	Langmuir			Freundlich			Dubinin–Raduskevich–Kaganer		
			*q*_max_ (mg g^−1^)	*b* (L mg^−1^)	*R* ^2^	*n*_f_	*k*_f_ (mg^1−*n*_f_^ L^*n*/*g*^)	*R* ^2^	*q*_DRK_ (mg g^−1^)	*E*_s_ (J mol^−1^)	*R* ^2^
Rac-MET	L27	633 ± 14	582 ± 31	0.835 ± 0.040	0.964	4.03 ± 0.10	266 ± 14	0.977	571 ± 25	16 846 ± 830	0.964
	AQ630	423 ± 10	402 ± 22	3.17 ± 0.10	0.753	9.65 ± 0.50	279 ± 11	0.936	407 ± 21	20 063 ± 1000	0.825
	S21	485 ± 5	570 ± 32	0.097 ± 0.002	0.991	3.25 ± 0.30	140 ± 8	0.968	506 ± 27	10 981 ± 540	0.992
	R-KC	1178 ± 9	1023 ± 55	40.5 ± 3.0	0.909	8.30 ± 0.30	760 ± 26	0.987	1046 ± 55	26 149 ± 1200	0.929
S-MET (60%)	L27	580 ± 11	823 ± 21	0.149 ± 0.020	0.890	3.03 ± 0.20	212 ± 12	0.888	749 ± 31	12 112 ± 500	0.890
	AQ630	547 ± 7	470 ± 24	5.58 ± 0.40	0.809	6.96 ± 0.40	267 ± 14	0.924	473 ± 25	21 115 ± 900	0.821
	S21	463 ± 3	560 ± 11	0.076 ± 0.001	0.964	2.93 ± 0.10	116 ± 7	0.943	482 ± 26	10 437 ± 500	0.968
	R-KC	970 ± 8	920 ± 41	1.85 ± 0.10	0.898	6.49 ± 0.30	536 ± 24	0.903	928 ± 47	18 606 ± 950	0.939
S-MET (100%)	L27	940 ± 16	1043 ± 53	0.279 ± 0.010	0.987	3.03 ± 0.10	329 ± 16	0.965	985 ± 52	13 662 ± 700	0.987
	AQ630	778 ± 12	785 ± 41	0.18 ± 0.03	0.965	3.51 ± 0.20	237 ± 9	0.997	724 ± 35	12 715 ± 610	0.954
	S21	290 ± 4	271 ± 12	3.50 ± 0.40	0.880	7.41 ± 0.30	162 ± 9	0.830	285 ± 21	19 463 ± 980	0.978
	R-KC	1110 ± 12	1134 ± 51	0.763 ± 0.040	0.729	6.67 ± 0.40	662 ± 31	0.737	1123 ± 53	16 280 ± 800	0.730

L27 and AQ630 were selected to study the influence of the solution pH during adsorption, due to its different acidic properties (Table 2). The natural pH of the Rac-MET was *ca.* 6 and the adjustment to 2 (with HCl 0.1 M) was made to analyse the effect of the media acidification. No significant differences were obtained between both solutions pH (Fig. S5 ESI†) indicating that the adsorption of Rac-MET was not affected by the pH, discarding the electrostatic interactions as expected due to the non-polar character of the molecule. Indeed the adsorption would occur through π–π dispersive interactions between the aromatic ring of the herbicide and π-electrons of the adsorbent structure. Similar observation was made for the adsorption of *S*-metolachlor on mesoporous resins.^27^

### 3.3 Adsorption of *S*-metolachlor: kinetics and isotherms

**Fig. 5:**
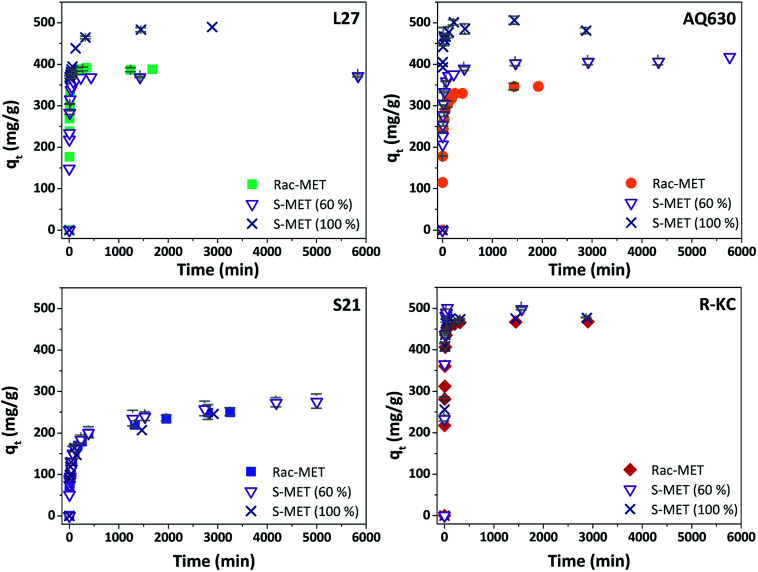
Evolution of Rac-metolachlor, *S*-metolachlor (60%) and *S*-metolachlor (100%) adsorption as a function of the contact time at 25 °C for the four adsorbents. The error bars represent the standard deviation.

Fig. 5 and S6 ESI† show the kinetic profile obtained for the four studied adsorbents employing different enantiomeric mixtures of metolachlor (Rac-MET, S-MET (60%) and S-MET (100%)). Interesting results can be observed as a function of the employed adsorbent. In the case of S21 and R-KC no significant differences were observed as a function of the enantiomer proportion indicating that, for these adsorbents, the adsorption is not selective. However, for the L27 and AQ630 different adsorption capacity was observed as a function of enantiomer's proportion. For L27, when the pure *S*-enantiomer is employed the adsorption at equilibrium increases from 390 to 485 mg g^−1^, while between 50% and 60% of *S*-enantiomer no difference was observed (Fig. 5, S6 ESI† and Table 3). For AQ630 the selectivity is more pronounced suffering an increase from 340 to 405 mg g^−1^ when the *S*-concentration is increased in 10% and when the pure *S*- is employed, the adsorption goes up to 505 mg g^−1^. In spite of the change in the adsorbed amount at equilibrium time, the shape of the adsorption kinetics was not suffered any modification, indicating that the followed adsorption process was not varied as a function of the enantiomer's proportion adsorbed. Indeed, in all cases, the kinetic adsorption followed a pseudo-second order model as is indicated by the fitting parameters (Table 3).

**Fig. 6:**
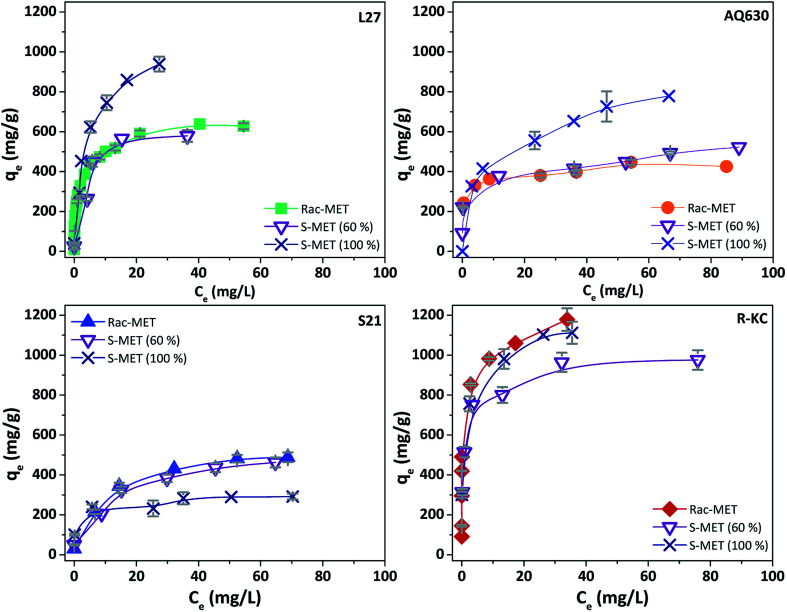
Experimental isotherms of Rac-metolachlor, *S*-metolachlor (60%) and *S*-metolachlor (100%) at 25 °C for the four adsorbents. The error bars represent the standard deviation.

To further investigate this behaviour, Fig. 6 and S7 ESI† show the adsorption isotherms for all studied adsorbents employing the three enantiomer's mixtures. As was previously observed on the kinetics adsorption, important increase on the adsorption capacity was observed when the *S*-enantiomer is employed for adsorption on L27 and AQ630 materials. On the other hand, for S21 and R-KC adsorbents, slightly differences were also observed at high concentration range.

To understand the adsorption process of the aromatic compounds, it is necessary to take into account the molecular dimension and orientation of the herbicides.^28^ In the case of enantiomers, since they have the same molecular weight, the different orientation could be the reason of the differences on the adsorption capacities. Metolachlor presents two chiral elements, an asymmetrically-substituted carbon atom, and a chiral axis (C–N bond). The presence of the chiral carbon atom results in two enantiomers (*R*- and *S*-). Additionally, the carbonyl group can be located away or toward the phenyl ring (positioned perpendicularly respect to the amidyl group) rendering four atropisomers: *aS* 1′*S*; *aR* 1′*S*; *aS* 1′*R* and *aR* 1′*R* (Fig. S8 ESI†).^9,29,30^ The conformation in which the molecule is adsorbed is important to be considered since it could induce changes on the electronic cloud and consequently it will have influence in the π–π interactions responsible for the adsorption process.

The samples that showed the highest effect of the enantioselective adsorption (L27 and AQ630) are the activated carbons with higher mesopore volume (Table 2) showing the percentage of occupied microporous surface close to 100% (see Section 3.2). This fact indicates that the founded differences on the adsorption capacity as a function of adsorbed enantiomer at samples L27 and AQ630 are mainly caused by the accessibility to the larger pores. According to our findings, the S-MET could adopt the adequate conformation to be greater retained in the mesoporous surface, compared with the R-MET, favouring the dispersive interactions between the herbicide and the activated carbon surface and increasing the adsorption capacity obtained.

## 4. Conclusions

Four activated carbons have been employed to study the adsorption of metolachlor herbicide frequently detected in both surface water and groundwater. The adsorbents presented different textural properties in terms of BET area, micro and mesoporous volumes and similar chemical composition. The adsorption uptakes revealed that the mesoporous structure combined with the presence of microporosity is a determining factor in the adsorption capacity. The R-KC carbon presented the higher adsorption of the herbicide associated to its high BET area and the micro/mesoporous network. The acidification of the aqueous media did not render changes on the adsorption capacities pointing out that the adsorption of metolachlor is mainly occurring through non-electrostatic interactions. Interestingly, we have observed an enantioselective adsorption as a function of used carbon material. In the case of L27 and AQ630 adsorbents, the enantiomer *S*-metolachlor is greater adsorbed indicating that the *S*-isomer of the molecule adopts the adequate conformation to be retained in the mesoporous structure. This finding supposes an interesting achievement since the S-MET is the most active enantiomer as herbicide and the selective adsorption will allow the recover, separation and purification of the herbicide.

## Conflicts of interest

There are no conflicts to declare.

## Supplementary Material

RA-010-D0RA07745C-s001
