# Supplementary material for: Study of enantioselective metolachlor adsorption by activated carbons
Source: RSC Adv. 2020 Nov 5;10(66):40321–8. doi: 10.1039/d0ra07745c (PMC9057506; doi:10.1039/d0ra07745c)
Supplement: RA-010-D0RA07745C-s001 [file RA-010-D0RA07745C-s001.pdf]

Electronic Supplementary Material (ESI) for RSC Advances.
This journal is © The Royal Society of Chemistry 2020

Study of enantioselective metolachlor adsorption by activated carbons

Alicia Gomis-Berenguer*[a], Isabelle Laidin[b], Sophie Renoncial[b] and Benoît Cagnon*[a]

[a]Interfaces, Confinement, Matériaux et Nanostructures-ICMN, UMR 7374-CNRS, Université d'Orléans, 1B, rue de la Férollerie, 45071 Orléans Cedex 2, France.

[b]JACOBI carbons, 15 Route de Foëcy, 18100 Vierzon, France.

ELECTRONIC SUPPLEMENTARY INFORMATION

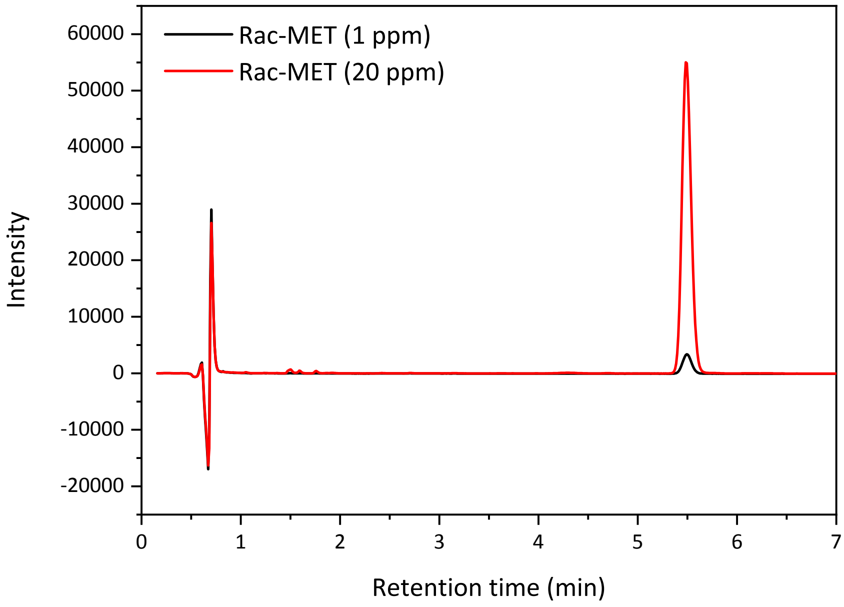

Figure S1. Example of chromatogram obtained for Rac-Metolachlor (1 and 20 ppm) at 230 nm.

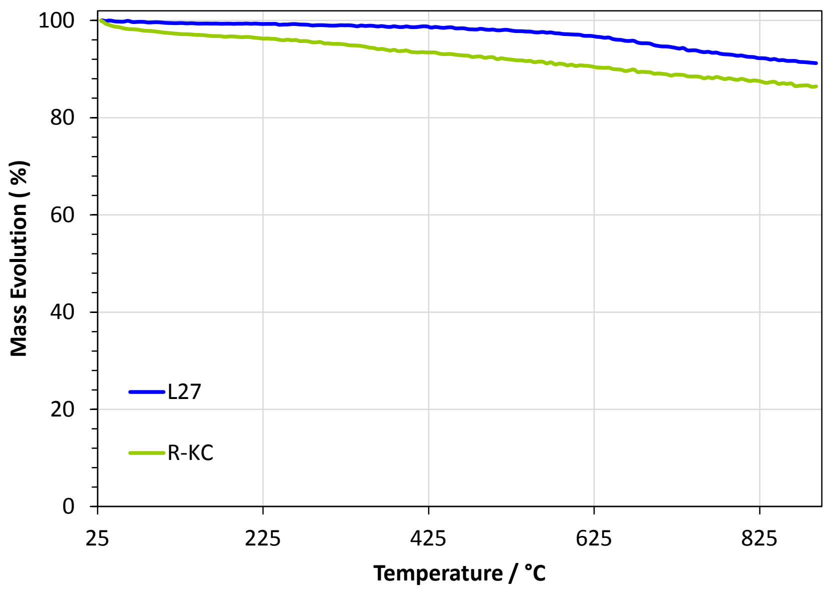

Figure S2. Thermogravimetric profile of R-KC and L27 adsorbents obtained under Ar flow (50 mL/min, 10 °C/min).

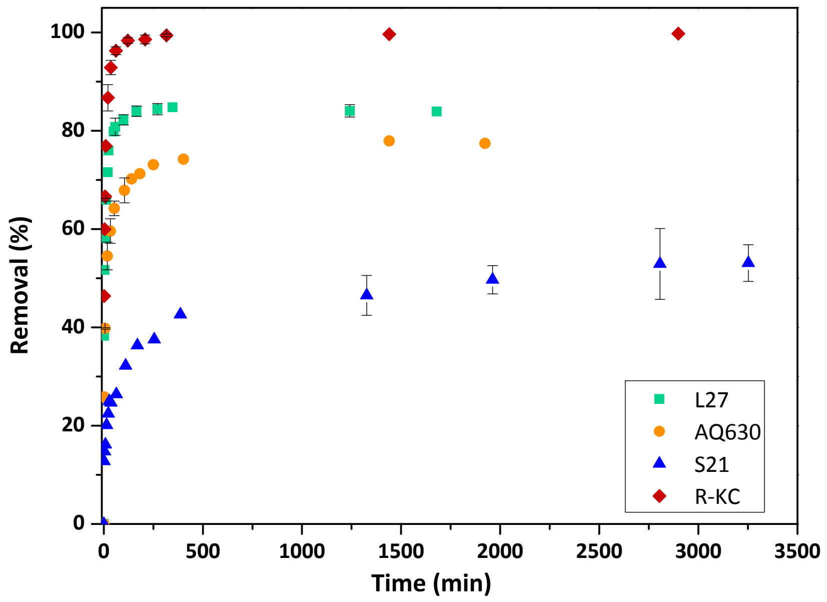

Figure S3. Evolution of Rac-Metolachlor adsorption in removal percentage as a function of the contact time at 25 °C for the four adsorbents. The error bars represent the standard deviation.

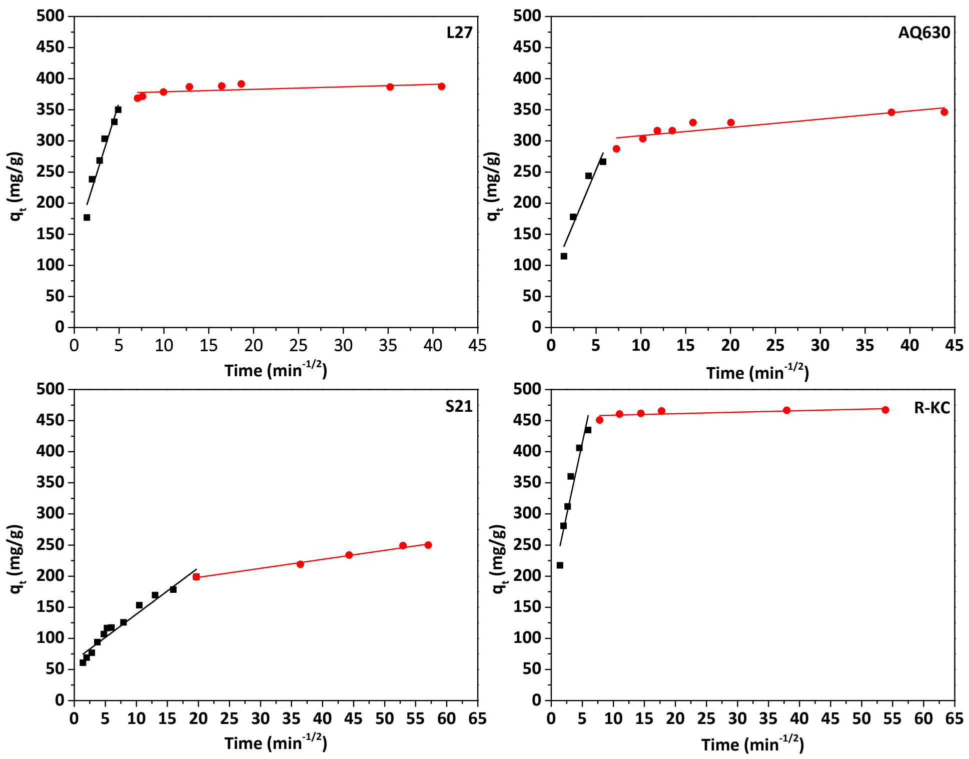

Figure S4. Intraparticle diffusion for Rac-Metolachlor on L27, AQ630, S21 and R-KC.

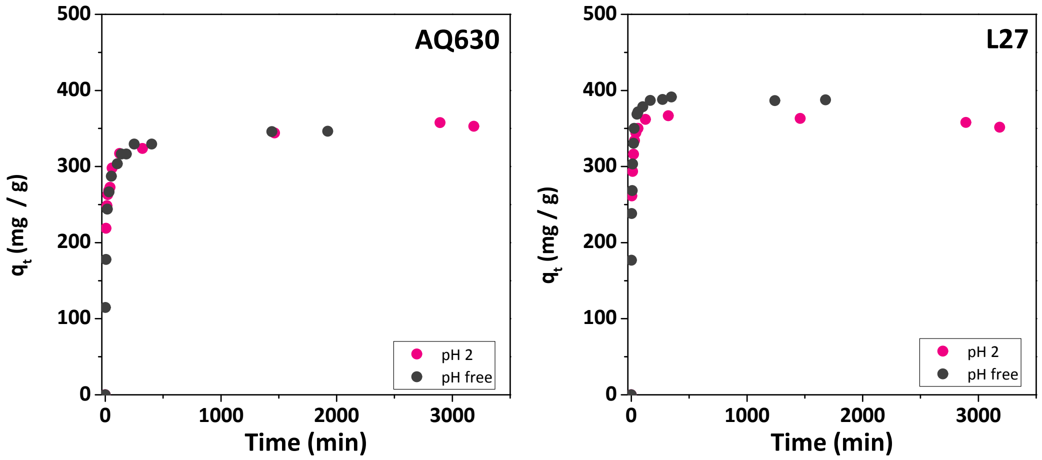

Figure S5. Adsorption kinetics of Rac-Metolachlor on AQ630 (left) and L27 (right) in aqueous solution at pH free and pH 2.

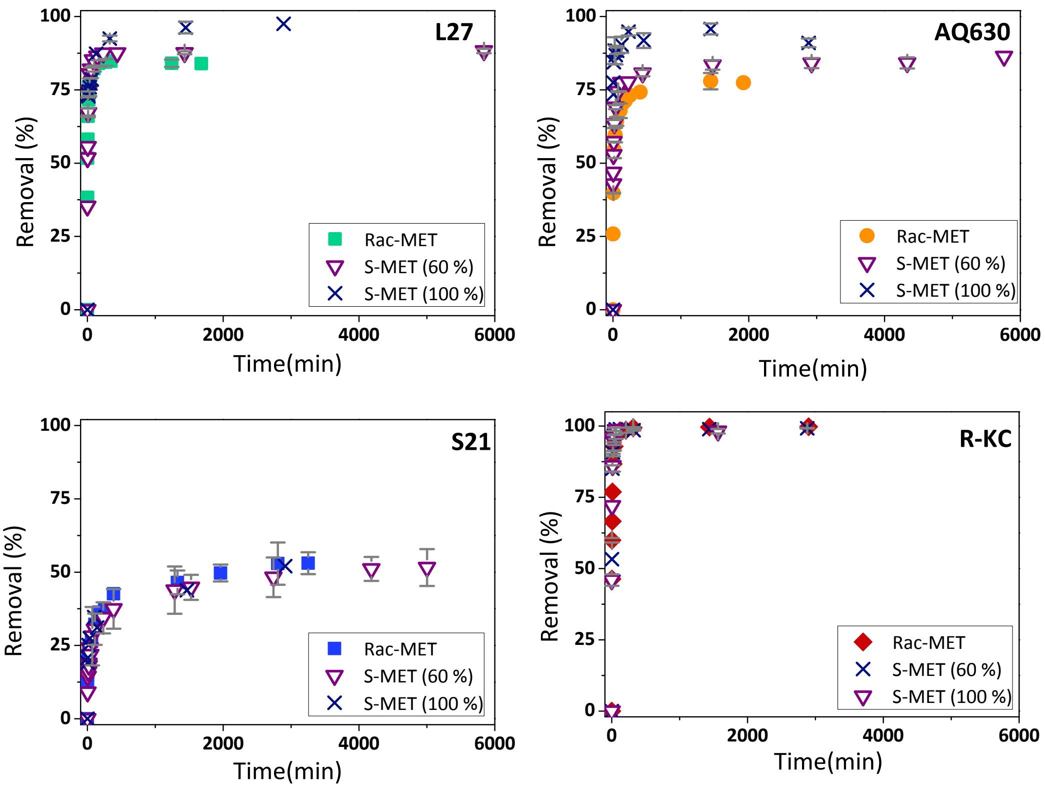

Figure S6. Evolution of Rac-Metolachlor, S-Metolachlor (60 %) and S-Metolachlor (100 %) removal efficiency as a function of the contact time at 25 °C for the four adsorbents. The error bars represent the standard deviation.adsorption in removal percentage.

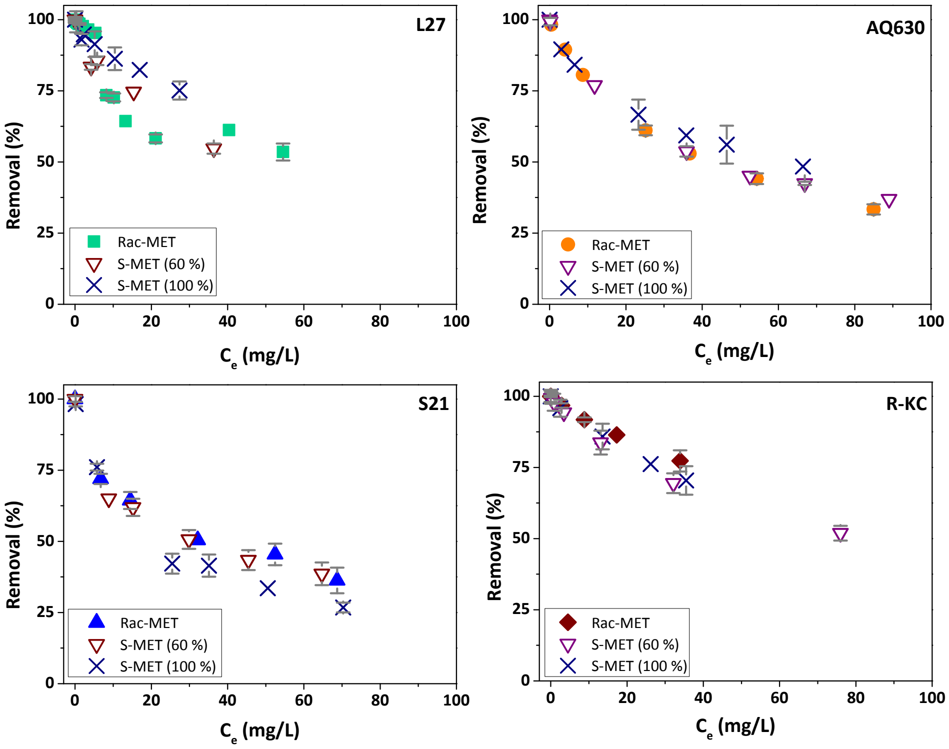

Figure S7. Experimental efficiency removal percentage of Rac-Metolachlor, S-Metolachlor (60 %) and S-Metolachlor (100 %) at 25 °C for the four adsorbents. The error bars represent the standard deviation

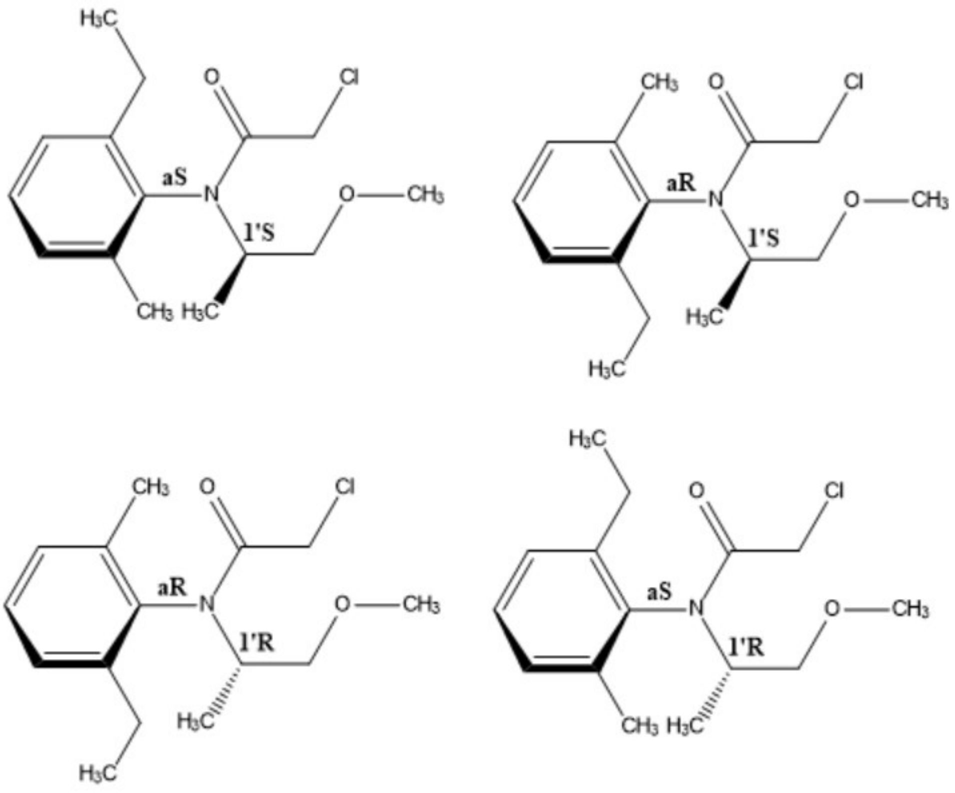

Figure S8. Atropisomers of Metolachlor molecule.
